# Frequency of Congenital Anomalies in the Brazilian Midwest and the Association with Maternal Risk Factors: Case-control Study

**DOI:** 10.1055/s-0040-1709692

**Published:** 2020-04

**Authors:** Carolina Leão de Moraes, Natália Cruz e Melo, Waldemar Naves do Amaral

**Affiliations:** 1 Faculty of Medicine, Department of Obstetrics and Gynecology, Hospital das Clínicas, Universidade Federal de Goiás, Goiânia, GO, Brazil; 2 Escola Paulista de Medicina, Universidade Federal de São Paulo, SP, Brazil

**Keywords:** congenital anomalies, ultrasound, prenatal, prenatal diagnosis, risk factors, anomalias congênitas, ultrassom, pré-natal, diagnóstico pré-natal, fatores de risco

## Abstract

**Objective** To evaluate the frequency of structural congenital anomalies (CAs) in the midwest of Brazil and its association with maternal risk factors.

**Methods** This was a prospective, observational, case-control study based on a hospital population. Pregnant women attended at a fetal medicine service in Brazil were analyzed in the period from October 2014 to February 2016.A total of 357 pregnant women were included, 223 of whom had fetuses with structural anomalies (group case), and 134 of whom had structurally normal fetuses (control group). The clinical history was made previous to prenatal consultation, and the diagnosis of the structural CA was performed through ultrasound.

**Results** A frequency of 64.27% (*n* = 223) of pregnant women with fetuses with structural anomalies was observed. The most frequent structural CAs were those of the central nervous system (30.94%), followed by anomalies of the genitourinary system (23.80%), and, finally, by multiple CAs (16.60%). The background of previous children with CAs (odds ratio [OR]: 3.85; *p* = 0.022), family history (OR: 6.03; *p* = < 0.001), and consanguinity between the progenitors (OR: 4.43; *p* = 0.034) influenced the occurrence of structural CA.

**Conclusion** The most frequent CAs are those of the central nervous system, followed by those of the genitourinary system, and then multiple anomalies. The maternal risk factors that may have influenced the occurrence of structural CA were previous children with CA, family history, and consanguinity among the parents.

## Resumo

**Objetivo** Avaliar a frequência de anomalias congênitas (ACs) estruturais no centro-oeste brasileiro e a associação com fatores de risco maternos.

**Métodos** Estudo prospectivo, observacional, caso-controle, baseado em uma população hospitalar. Foram analisadas gestantes atendidas em um serviço de medicina fetal no Brasil, no período de outubro de 2014 a fevereiro de 2016. Foram analisadas 357 gestantes, dentre as quais 223 tiveram fetos com ACs estruturais (grupo controle) e 134 tiveram fetos estruturalmente normais (grupo controle). A história clínica foi feita antes da consulta de pré-natal, e o diagnóstico da AC estrutural foi realizado por ultrassonografia.

**Resultados** Observou-se uma frequência de 64,27% (*n* = 223) de gestantes com fetos com ACs estruturais. As ACs estruturais mais frequentes foram as do sistema nervoso central (30,94%), seguidas das anomalias do sistema gênito-urinário (23,80%), e, por fim, das ACs múltiplas (16,60%). Antecedentes de crianças com AC (razão de probabiliade [RP]: 3,85; *p* = 0,022), antecedentes familiares (RP: 6,03; *p* = < 0,001), e consanguinidade entre os grupos progenitores (RP: 4,43; *p* = 0,034) influenciaram a ocorrência de AC estrutural.

**Conclusão** As ACs mais frequentes foram as do sistema nervoso central, as do sistema gênito-urinário, e as ACs múltiplas. Os fatores de risco maternos que podem ter influenciado a ocorrência de AC estrutural foram antecedentes de crianças com AC, história familiar, e a consanguinidade entre os pais.

## Introduction

Congenital anomalies (CAs) are among the main causes of death in children under 5 years of age.^1^ It is estimated that between 3 and 7% of children are born with birth defects worldwide,^2^ and that ∼ 270,000 newborns die during the first 28 days of life every year.^2^ ^3^ In developed countries, CA is the leading cause of death in children, while in developing countries, mortality by CA is still not considered a public health problem.^4^ However, with the control of infections and diseases of nutritional deficiency, there is a tendency to reduce infant mortality for these reasons; thus, congenital malformations have become important causes of perinatal mortality in countries such as Brazil.^5^ ^6^ Currently, ∼ 60% of the etiology of CAs in human beings are not elucidated. However, in around 25% of CAs, the causes seem to be multifactorial, reflecting a complex interaction of known and unknown genetic and environmental factors, including sociocultural, racial, and ethnic variables.^7^ In Brazil, there is a shortage of data on the incidence of CA and the associated maternal risk factors. The absence of comprehensive studies on CAs in Brazil justifies a prospective study case control that aims to describe the frequency of structural CAs and the characteristics of pregnant women to determine possible risk factors associated with the structural CA. The results presented herein can help in the development of strategies to improve the management, genetic counseling, and rehabilitation of patients with CA as well as the taking of public health measures to determine risk factors.

## Methods

This was a prospective, observational, case-control study based on a hospital population. Pregnant women attended at a fetal medicine service in Brazil were analyzed in the period from October 2014 to February 2016.The research ethics committee of the institution approved the research with the number 808.377. Participants who responded to the questions asked during the interview and performed all the prenatal follow-up at the institution were included in the study. The collection of data was obtained through interview of the pregnant women, using a preform that contained personal and family history (maternal age, maternal ethnicity, previous children with CA, CA family history, and consanguinity) data. Data on previous obstetric history (number of previous pregnancies and prior abortions) were also verified. The presence of structural CA and its classification was confirmed by prenatal ultrasound evaluation by a fetal medicine specialist in. After the monitoring of ultrasounds, the pregnant women were categorized in the case or control groups. The case group was made up of pregnant women of fetuses with structural anomalies, and the control group by pregnant women whose fetuses did not have structural abnormalities. The pregnant women in the case group were accompanied by the main researcher in all the consultations performed after the diagnosis of CA. Thus, it was possible to update the information concerning the development of the fetus. The results of childbirth and newborns with structural anomaly were obtained by telephone contact with the pregnant women, in the computerized reports system, and, in the cases of childbirth performed in the hospital where the study was conducted, by consulting the medical file. The data were analyzed through descriptive statistics (average, standard deviation [SD], absolute frequency, relative frequency, median, confidence interval [CI]), Chi-squared tests, odds ratio, and the IBM SPSS Statistics for Windows version 22.0 software (IBM Corp., Armonk, NY, USA). Values of *p* < 0.05 were considered statistically significant.

## Results

In the investigation period, 357 pregnant women were sent for attendance at the institution. Of these, 62.46% (223/357) were pregnant with fetuses with structural anomalies (case group), and 37.54% (134/357) were pregnant with structurally normal fetuses (control group). The average age of pregnant women in the case group was 25.73 years, and, in the control group, it was 25.39 years. Table 1 describes the study population in detail.

**Table 1:** Description of sociodemographic and obstetric data of pregnant women

Variables	Population			
	Case		Control	
	n	%	n	%
Maternal age				
≤ 18	32	14.34%	19	14.18%
19–24	70	31.40%	46	34.33%
25–30	69	30.94%	34	25.37%
31–36	41	18.39%	26	19.40%
≥ 37	11	4.93%	9	6.72%
Ethnicity				
White	46	20.62%	45	33.58%
Brown	128	57.40%	62	46.27%
Black	45	20.20%	27	20.15%
Indigenous	4	1.80%	0	–
Nr. of gestations				
Primigravida	92	41.26%	46	34.33%
Multigravida	131	58.74%	88	65.67%
One previous gestation	*68*	*51.91%*	*42*	*47.73%*
Two previous gestations	*38*	*29.00%*	*33*	*37.50%*
* ≥* Three previous gestations	*25*	*19.09%*	*13*	*14.77%*
History of abortion				
No	180	80.72%	120	89.55%
Yes	43	19.28%	14	10.45%
Previous gestation	*12*	*27.91%*	*5*	*35.72%*
In one of two previous pregnancies	*18*	*41.86%*	*2*	*14.28%*
In one of ≥ three previous pregnancies	*13*	*30.23%*	*7*	*50.00%*
Children with CA				
No	205	91.93%	131	97.76%
Yes	18	8.07%	3	2.24%
Previous gestation	*2*	*11.11%*	2	66.67%
In one of two previous pregnancies	*10*	*55.56%*	0	–
In one of ≥ three previous pregnancies	*6*	*33.33%*	1	33.33%
Family history of CA				
No	148	66.37%	124	91.94%
Yes	75	33.63%	10	8.06%
Parents with CA	*7*	*9.33%*	1	*10.00%*
Brothers or grandmothers with CA	*23*	*30.67%*	4	*40.00%*
Uncles and grandmothers with CA	*10*	*13.33%*	0	*-*
Uncles, grandmothers, and cousins with CA	*22*	*29.33%*	5	*50.00%*
CA in several relatives	*13*	*17.33%*	0	*-*
Consanguinity				
No	209	93.72%	132	98.51%
Yes	14	6.28%	2	1.49%
Total	223	100%	134	100%

Abbreviations: %, frequency; CA, congenital anomaly; mean, arithmetic mean; n, sample.

The most frequently diagnosed CAs were anomalies of the central nervous system (CNS) (30.94%; *n* = 69), followed by anomalies of the genitourinary system (GUSs) (23.80%; *n* = 53), and, finally, by multiple congenital anomalies (MCAs) (16.60%; *n* = 37). Table 2 demonstrates the distribution of major structural CAs, according to topography and type of lesion. In addition, other abnormalities, such as abdominal (8.52%; *n* = 19), cardiovascular (6.30%; *n* = 14), and lymphatic system (5.82%; *n* = 13), among others (8.02%; *n* = 18), were observed.

**Table 2:** Distribution of main structural congenital anomalies according to topography and type of lesion

Congenital anomalies	n	%
Central nervous system		
Hydrocephalus	23	33.33%
Anencephaly	16	23.20%
Meningocele	7	10.14%
Others	23	33.33%
Total	69	100%
Genitourinary system		
Renal dysplasia	20	37.73%
Hydronephrosis	13	24.53%
Pyelectasis	12	22.64%
Others	8	15.10%
Total	53	100%
Multiple anomalies		
Craniofacial and limbs	13	35.14%
Craniofacial and cardiac	9	24.32%
Craniofacial and digestive	6	16.22%
Others	9	24.32%
Total	37	100%

Abbreviations: %, frequency; n, sample.

When comparing the case group with the control group, the data analysis revealed a statistically significant difference in relation to the CA family history (*p* < 0.001, CI: 3.12–12.67), indicating that pregnant women with relatives who have structural CAs have 6.03 more chance of develop fetuses with structural CAs. Patients with previous children with CAs (*p* = 0.022) and consanguinity (*p* = 0.034) also showed a statistically significant difference between the groups (Table 3).

**Table 3:** Distribution of cases of fetal evaluation according to the characteristics of pregnant women attended at a fetal medicine service

Variables	Population						
	Case		Control		OR	95%CI	*p*-value
	n	%	n	%			
Maternal age							
< 35	21	90.42%	12	80.96%	–	0.50–2.22	0.884
≥ 35	202	90.58%	122	91.04%			
Nr. of gestations							
Primigravida	92	41.26%	46	34.33%	–	0.86–2.10	0.193
Multigravida	131	58.74%	88	65.67%			
Previous children with CA							
Yes	18	8.07	3	2.24%	3.85	1.11–13.27	0.022
No	205	91.93	131	97.76%			
Family history of CA							
Yes	75	33.63%	10	8.06%	6.03	3.12–12.67	< 0.001
No	148	66.37%	124	91.94%			
Consanguinity							
Yes	14	6.28%	2	1.49%	4.43	0.99–19.76	0.034
No	209	93.72%	132	98.51%			
Total	223	100%	134	100%			

Abbreviations: %, frequency; 95%CI, 95% confidence interval; CA, congenital anomaly; n, sample; OR, odds ratio.

## Discussion

During the investigation period, a frequency of 62.46% of pregnant women with fetuses carrying structural anomalies was observed. The CNS, GUS, and MC anomalies were the most frequent ones. Indian studies showed similar results.^8^ ^9^ ^10^

Differently, other studies report higher frequency of CAs of the cardiovascular system.^5^ ^11^ ^12^ ^13^ On the other hand, the higher frequency of CNS has been reported in several studies in Iran,^14^ Japan,^15^ Pakistan,^16^ ^17^ China,^18^ Nigeria,^19^ Tanzania,^20^ and India.^8^ ^9^ ^10^

The etiology of CNS anomalies is multifactor and involves complex interactions between genetic and environmental factors, constituting one of the most common congenital defects.^9^ ^21^ ^22^ Among the anomalies of the CNS observed in this study, hydrocephalus and anencephaly were the most reported changes, which is similar to other studies that also reported hydrocephalus^8^ ^14^ ^17^ ^23^ and the anencephaly^8^ ^15^ ^17^ ^24^ among the most common malformations.

The data in this study indicated that the occurrence of fetal malformation in one or more family members is associated with the development of CAs in the current gestation. Pregnant women who have a family history of CAs are 6.03 times more likely to develop fetuses with some structural anomaly. the literature data already highlighted this association.^8^ ^23^ Correia et al^25^ revealed that 16% of families with registered cases of fetal malformations in Portugal had one or more family members with CAs. In addition, studies indicate that some specific CAs, such as those of the kidney and heart, have the potential to aggregate into families.^26^ ^27^

In this study, the pregnant women who have had children with some CA presented 3.85 times more chance of having other children with malformations. These data are similar to the results of Lie et al,^28^ which showed that mothers who already had a child with CA would have a 2.4 times greater risk of having a second gestation affected when compared with a pregnant woman without a history of CA occurrence. Marwah et al^8^ observed higher frequency of malformations in pregnant women who had already had children with CA. Thus, possibly, there is a strong tendency of recurrence of specific defects in the same family, indicating the persistence of a causal factor.

Regarding consanguinity, it was verified that consanguineous parents presented 4.43 times more chance of having children with anomalies than parents with no degree of kinship. These data are concordant with other studies that show a positive association between CA and consanguineous parents.^8^ ^9^ ^11^ ^23^ ^29^ However, Hatibaruah and Hussain^30^ found no relation between consanguinity and CA, and Neira et al^31^ did not observe cases of consanguinity among the parents of malformed newborns.

Maternal age is considered an important parameter in the birth of a fetus with CA and patients aged <20 or >40 years old may showed increased risk of having children with certain birth defects.^32^ However, in our study, the correlation between maternal age and CA was not evident (*p* = 0.884). Similar to our findings, the study by Francine et al.^11^ et al also did not report the occurrence of this association. Despide, some studies have reported the association of increased maternal age and the occurrence of CA.^8^ ^15^

There are few studies in the literature that evaluate number of pregnancies as a risk factor for the occurrence of CA. Our study found no differences between the occurrence of AC between and multigravida and primigravida. But, we can verify a higher frequency of CA in multigravida and this result is in agreement with other data in the literature.^8^ ^16^ ^30^ While, other studies have reported a higher frequency of CA in primigravida.^9^ ^30^ ^31^ ^32^ ^33^ Thus, the data still do not conclude how parity can influence the occurrence of CA.

The differences between studies can be reflected in different racial, ethnic, and social factors in various regions of the world. Other justifications for these variations include the different study methodologies used for sampling, accessibility, and use of advanced diagnostic techniques, which improve the early and correct detection of CAs.^14^

The current study presents some limitations. First of all, the collected data were from a fetal medicine service, and the prevalence showed may be greater than that of the general population. Because genetic tests are not offered by the institution, tests such as karyotype, that could prove the influence of parental genetics in the occurrence of structural CA, were not performed. However, we recognize the importance of such tests. Despite the aforementioned limitations, we emphasize the importance of this work, mainly because it is prospective and because it presents the reality from the midwest of Brazil.

## Conclusion

In the present study's population, a higher frequency of CNS, GUS, and MC anomalies was observed. The maternal risk factors that may have influenced the occurrence of structural CAs were previous children with CA, family history, and consanguinity. The results related here are important for the development of strategies to improve the management, genetic counseling, and rehabilitation of patients with CA as well as for the taking of public health measures for risk factors.
